# Blockchain Private File Storage-Sharing Method Based on IPFS

**DOI:** 10.3390/s22145100

**Published:** 2022-07-07

**Authors:** Peng Kang, Wenzhong Yang, Jiong Zheng

**Affiliations:** 1 College of Information Science and Engineering, Xinjiang University, Urumqi 830046, China; 18290639804@163.com (P.K.); zhengjiong@xju.edu.cn (J.Z.); 2 Key Laboratory of Multilingual Information Technology in Xinjiang Uygur Autonomous Region, College of Information Science and Engineering, Xinjiang University, Urumqi 830046, China

**Keywords:** blockchain, decentralization, NDN, IPFS, shared storage

## Abstract

Under the current national network environment, anyone can participate in publishing. As an important information resource, knowledge files reflect the workload of publishers. Moreover, high-quality knowledge files can promote the progress of society. However, pirated inferior files have the opposite effect. At present, most organizations use centralized servers to centrally manage the knowledge files released by users. In addition, it is necessary to introduce an untrusted third party to examine and encrypt the contents of files, which leads to an opaque process of file storage transactions, tampering with intellectual copyright, and the inability to have consistent systems of file management among institutions due to the lack of uniform standards for the same intellectual files. The purpose of this paper is to ensure the safe storage of knowledge files on the one hand and to realize efficient sharing of copyrighted files on the other hand. Therefore, this paper combines NDN (Named Data Network) technology with a distributed blockchain and an Interplanetary File System (IPFS) and proposes a blockchain knowledge file storage and sharing method based on an NDN. The method uses the NDN itself for the file content signature and encryption, thereby separating the file security and transmission process. At the same time, the method uses a flexible NDN reverse path forwarding and routing strategy, combining an IPFS private storage network to improve the safety of the encrypted data storage security. Finally, the method takes advantage of all participating nodes consensus and shares files in the synchronized blockchain to ensure traceability. This paper introduces the structure and principles of the method and describes the process of file upload and transfer. Finally, the performance of the method is compared and evaluated, and the advantages and disadvantages of the method and the future research direction are summarized.

## 1. Introduction

In the current environment with large amounts of data, the problem of data storage and access is commonplace. When choosing to store or share time-sensitive files, selecting a mobile hard disk is a cumbersome process, with the authenticity, security and confidentiality of the third-party storage platform often uneven. Moreover, at the same time, the isolation between data and the cost of the data center in the process of distribution pose challenges. In addition, the management standards of different platforms are not unified, the problems of inferior duplicate files and malicious charges cannot be solved, and the source of junk malicious files cannot be traced and deleted effectively. Therefore, there is an urgent need for a safe and transparent technology that can efficiently store and forward files and ensure the authenticity of files to solve the problems existing in the field of intellectual copyrighted files. At present, the number of blockchain-based applications addressing the needs of government affairs, financial services, electronic bills, product traceability and other fields has increased significantly, and the process of network data distribution is timely. In the face of many challenges, such as security, an efficient content distribution technology and application scheme will greatly promote the development of network technology.

Distributed shared accounts originated in Bitcoin [1]. The blockchain is maintained by all the nodes in the system as a database to record all the transactions in the system. Blockchain is also used in a variety of other applications in many fields and industries, including healthcare, energy management and supply chains [2,3]. A blockchain is decentralized, tamper-proof, traceable, and attack-proof in building peer-to-peer (P2P) communication technology. A blockchain uses cryptography, distributed storage, a consensus mechanism, a smart contract and other technologies. In a P2P network, the communication quality and convergence speed of information are very important issues, and an NDN is used to optimize the inherent problems in a P2P network (such as mobility limitation, transmission semantic overload, double confirmation forwarding, etc.). An NDN is a content-centric network. Unlike traditional IP networks, data is published, requested, managed (modified, deleted, etc.), and accessed by data names. In the nature category of a blockchain, given the way the private chain’s authorized nodes work, the nodes trust each other, which is more conducive to privacy protection.

Knowledge files vary in size and contain various types of content. After they are stored directly on the blockchain and constantly updated for synchronization, blockchain data surges, and the chain storage pressure is too large. As a distributed file storage system, the interstellar file system can connect all the participating nodes to the unified storage system, which conveniently relieves the storage pressure on the blockchain itself. In addition, an IPFS solves the problem of data redundancy in the network by using content-based addressing.

The main work of this study can be summarized as follows:

1. An NDN-based blockchain knowledge file storage and transfer model is designed, and the relevant entities and roles involved in the model are introduced in detail. An NDN signature is used to encrypt the data content for storage, and the requested data are forwarded in a reverse path to improve the forwarding performance. Finally, the forwarding transaction process is synchronously stored on the blockchain to ensure the traceability of the knowledge file transfer process, thus ensuring the authenticity of the whole forwarding process.
2. Finally, the model also includes a local private IPFS network, which improves the efficiency of content publishers to upload NDN encryption knowledge files. In the model, the file content is stored in the private IPFS network, and the content owner and the corresponding hash value are stored on the blockchain to achieve efficient real storage.

The structure of this paper is as follows: Section 2 mainly introduces the relevant technologies and principles involved in the model; Section 3 introduces the process of file uploading and forwarding in detail through specific examples. In Section 4, the proposed model is evaluated by experimental simulation. Section 6 summarizes the advantages and disadvantages of the proposed model and further research work.

## 2. Related Theory

### 2.1. Blockchain

A blockchain is essentially a consensus system maintained by multiple parties, with decentralized participating nodes working together to maintain a reliable database. The working layers of a blockchain are shown in Table 1. Yang [4] summarized the development trend of network service architecture based on a blockchain. Smart contracts provide a complete blockchain technology solution and expand the application field of the blockchain. As the core technology service module of the blockchain, the smart contract often carries the core execution logic of the blockchain and is strong technical support for blockchain applications. Smart contracts are computer code running on distributed storage platforms that can be executed or run on a network of computers (typically the same working network that runs the blockchain) and may trigger ledger updates. Generally speaking, it is a computer protocol designed to propagate, validate or enforce contracts in an information-based manner and to allow trusted transactions to take place without third parties. These transactions are traceable and irreversible. In blockchain technology, a P2P network is adopted for message transmission and synchronization, which enhances the decentralization ability of the whole network and ensures the scalability and final synchronization consistency of nodes in the network.

### 2.2. NDN

**Figure 1:**
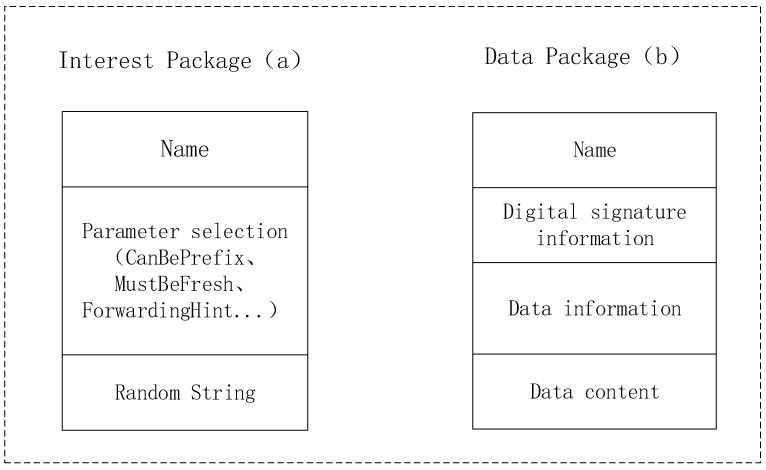
The interest packet (**a**) and the data packet (**b**).

The basic design principle of an NDN is based on the Internet [5], so it can directly use major IP services, such as a Domain Name System (DNS) and an interzone routing policy. It allows routers to track the status of packets and supports multipath forwarding. These features allow content to be cached in the router to meet future requests while enabling user mobility. Consumers send requests for the required data, called interest packets, which are forwarded to data producers by the NDN forwarding daemon (NFD) [6]. Each NFD also includes a forwarding policy module that determines whether, when and through which interfaces the received interest is forwarded [7]. In an NDN, the content packet consists of the data name, the encrypted content, and the signature with the publisher [8]. In addition, the NDN network has only two types of data packets, and the data packet format is shown in Figure 1.

**Figure 2:**
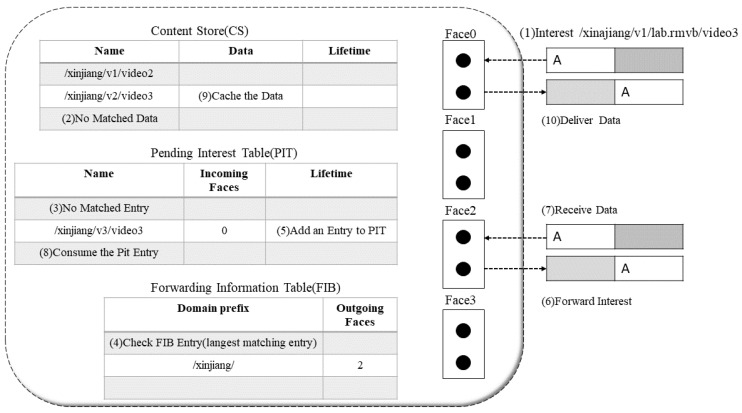
NDN forwarding model.

An NDN router consists of three main components: an undetermined interest table (PIT), a content store table (CS) and a forwarding information table (FIB). The PIT acts as a logger or recording system for recording packets that have been served by the router but whose contents have not yet reached the consumer. Moreover, the CS is a place to store data that has been submitted for a while. If other consumers request some interest and it is already in CS, then the storage mechanism can be very useful, and you can send the requested data immediately. Different from a PIT, an FIB, as a recorder or recording system [9], only stores the path where the content is located, and the communication forwarding process is shown in Figure 2. During a sharing process, the requester based on the name KP/NDN/blockchain.doc files sends a request packet, which then goes through the CS table the pit table and the FIB table looking up in turn. If there is a result in the CS, the CS forwards a content packet on a direct reverse path request route, otherwise the pit query continues. If there is a pit request and a corresponding interface after feedback from other nodes, the reverse path feedback and the content in CS is stored for a period of time for multiplexing and storage security aspects. The NDN network itself signs and encrypts content packets provided neither CS nor pit are present and finally found by the FIB. The FIB updates the request to content in potential nodes based on manually configured or named routing protocols, discarding the request package if the FIB is not present to indicate inaccessibility.

In general, compared with blockchain technology, a P2P network may cause message delay and duplication, low reuse rate of forwarded data, mobility restrictions and additional data security protection problems. An NDN has advantages in the following aspects:

1. Flexible routing and forwarding policies to balance network traffic by forwarding content in reverse paths;
2. Name, sign and encrypt the content, and the content-centered transmission mechanism optimizes the mobility limitation;
3. Routing naming cache mechanism ensures content security and transmission reuse rate.

### 2.3. IPFS

As a global point-to-point distributed file storage system, the Interplanetary File System (IPFS) was originally designed to optimize the current hypertext transfer protocol and then gather computer devices with the same file system together to form a huge distributed system [10]. At the same time, IPFS provides both a global node network and a more secure and efficient private shared network. This content search mechanism assigns a unique hash value to each file in the network based on the file content and maintains a distributed hash table to find the required file. For large storage files, IPFS will automatically slice files into multiple servers for storage and synchronously download and splice files upon request to speed up file access. In the model designed in this paper, an IPFS private storage network composed of three nodes was constructed to improve the overall efficiency of the model.

## 3. Methods

This section describes in detail the related processes, including publishers uploading knowledge files and requesters requesting content data.

1. Central authorization node: it is responsible for the registration review of new users in the model. According to the personal information provided by users, it records the personal information of the users that have passed the review and sends the registration information, including the login private key.
2. File publisher: This article refers to the file owner who encrypts the knowledge file and uploads it to an IPFS and private chain structure. These files will be used by the future owner to review the download or forward them to other peer requesters to receive the corresponding token reward. Publishers have the right to decide whether to accept access to downloads from other requesters.
3. File requester: The user sends a request to a file according to the NDN naming method. When a broadcast request is made within the network according to the attitude of the publisher, the knowledge file that is allowed to be forwarded is found through the IPFS and forwarded through the reverse path of the NDN’s daemon forwarding process router (NFD), and the forwarded content is saved in the passing routing table (CS) for a while for future access requests.
4. Private blockchain structure: This consists of three nodes: NDN node, block node, and IPFS node. It is responsible for uploading the publisher’s complete knowledge file content to the private IPFS network for preservation, storing the relevant IPFS hash value of the encrypted data content on the blockchain for traceability and recording the file flow information to the chain in the future when requester and publisher conduct a file transaction. File transfer performance can be optimized by using the NDN’s routing storage function and reverse path forwarding function.

**Figure 3:**
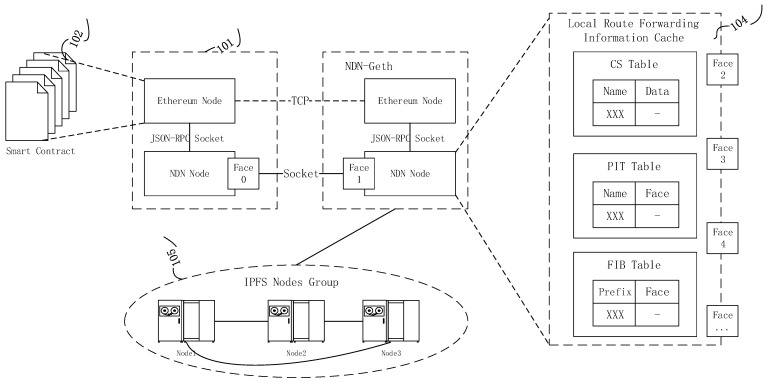
Data communication and forwarding process.

**Figure 4:**
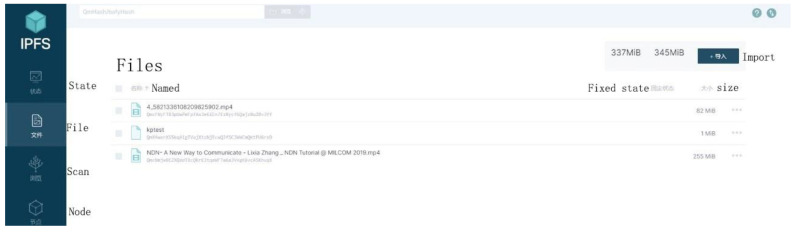
Interplanetary File System local file storage.

**Figure 5:**
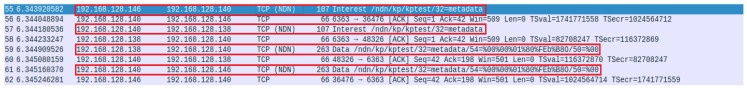
NDN file signature encryption result.

Figure 3 is the structure of the data storage communication of the model in this paper. It includes 102 forwarding rules controlled for established smart contracts; 101 is the underlying network structure of the NDN-Geth constructed; 104 is the process of file request search within the network; 105 is the local private IPFS network cluster constructed. Figure 4 shows the file storage results uploaded to the local IPFS, waiting for the consumer’s interest request (such as the request for kptest). If there are multiple kptest files, the request process will perform the longest name matching according to the NDN naming rules. During the upload process, the file content will also be signed and encrypted through the NDN. The results are shown in Figure 5.

### 3.1. Private File Save Process

1. The file publisher sends the signed and encrypted named content packet to the IPFS node group in the model through the NDN network.
2. One of the nodes of the private IPFS constructed in the model accepts the data uploaded by the file publisher and verifies the user’s identity through the user signature in the packet. After the authentication, the original ciphertext is uploaded to the private IPFS network for storage, and the unique hash value of the corresponding content is returned. The returned hash value is signed with the public key of the IPFS node.
3. Finally, the information received by the IPFS is simulated as a transaction and sent to the publisher’s private blockchain node for storage. The transaction includes the content hash corresponding to the file, signature hash, uploader information and the corresponding time stamp. The ciphertext corresponding file hash is authenticated and stored between the nodes by the blockchain, thus achieving the guarantee of untamperable authenticity under the premise that the file is stored securely.
4. After the publisher node stores and packages related transactions, it synchronously backs up transactions on other nodes of the private chain using the Gossip algorithm. Ultimately on the blockchain, the guarantee of true consistency of information storage of files between all participating nodes is achieved.

### 3.2. Private File Share Process

1. After the file requester passes the authentication of the central authorization node, the file requester obtains the user information of the authorized login, including the login private key.
2. The file requester sends the file access request of interest to the private chain validation cluster node, which includes the fixed interface information of the requester node and the name of the interested named file. After receiving the request, the NDN will route the interest request successively to the CS table, the PIT table and the FIB table described above for content query. After a preliminary judgment of the smart contract and forwarding strategy, if there is no relevant forwarding content in the local CS, the PIT will be queried. If there is a corresponding request record, the interface passed by the current interest packet to the matched record entry is added and waits for entering the FIB query. The FIB can be manually configured or updated based on the named routing protocol. If no forwarding rule exists in the FIB forwarding policy, interest request packets are discarded, and forwarding is not routed. If there is a forwarding rule, the corresponding interest request is forwarded to the corresponding content publisher, and the forwarding result is recorded in the PIT entry to forward the existing and allowed requests to the content publisher node. The publisher decides whether or not to forward the file to a specific requester, and if it agrees, it will forward the knowledge file in exchange for the corresponding token reward. If it does not agree, it will simply discard the file.
3. For the content that agrees to be forwarded for the first time, the content publisher node requests the IPFS node group. After verifying the identity of the publisher, the corresponding hash value is found by the name of the file submitted by the publisher, and the complete encrypted file data is retrieved from the model private IPFS network according to the hash result. The content publisher node forwards to the requesting node through the reverse path, and the corresponding file content and forwarding interface information is kept in the CS table and the PIT table for a while, respectively, to ensure the convenience of requesting again in the future, and the transaction process is stored on the private chain.
4. After receiving the encrypted original knowledge file from the content publisher, the requester uses the node information to verify the validity of the identity. After passing the authentication, the requester uses the content-related key to decrypt the ciphertext and obtain the complete knowledge file.

## 4. Results and Discussion

### 4.1. Network Performance

**Figure 6:**
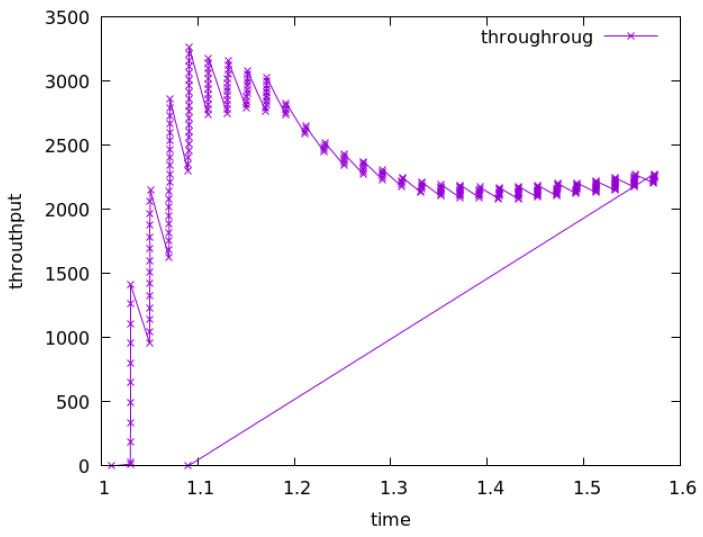
Throughput of traditional network.

**Figure 7:**
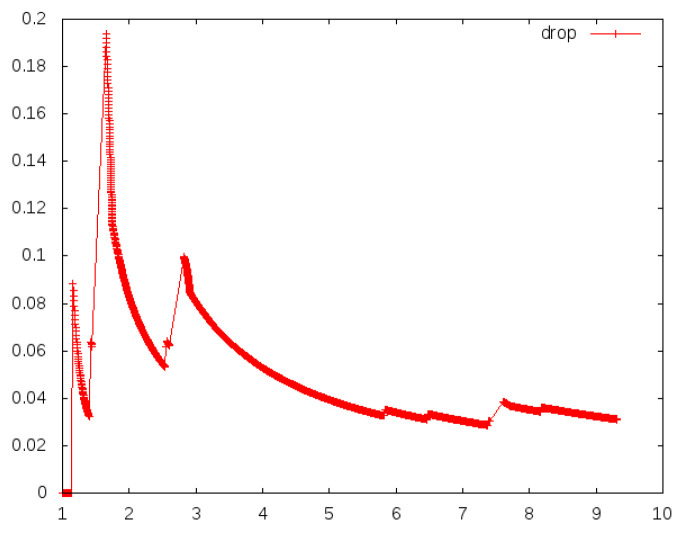
Drop of traditional networks.

**Figure 8:**
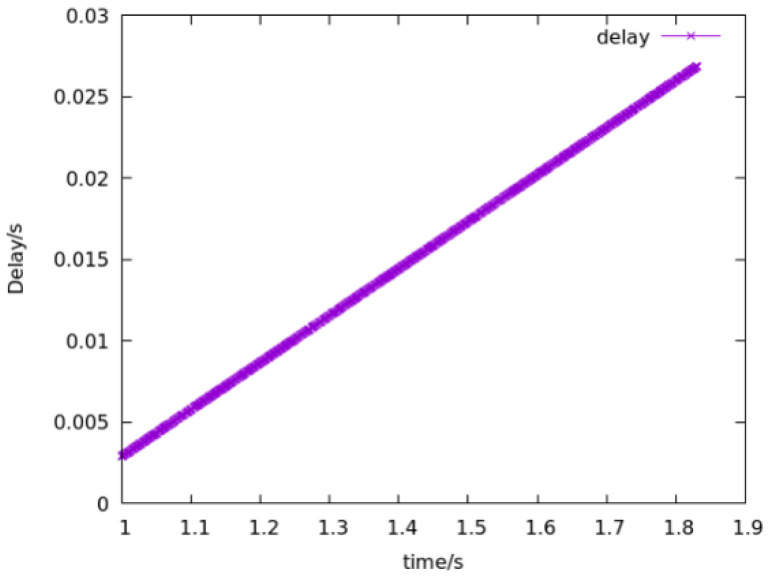
Delay of traditional network.

**Figure 9:**
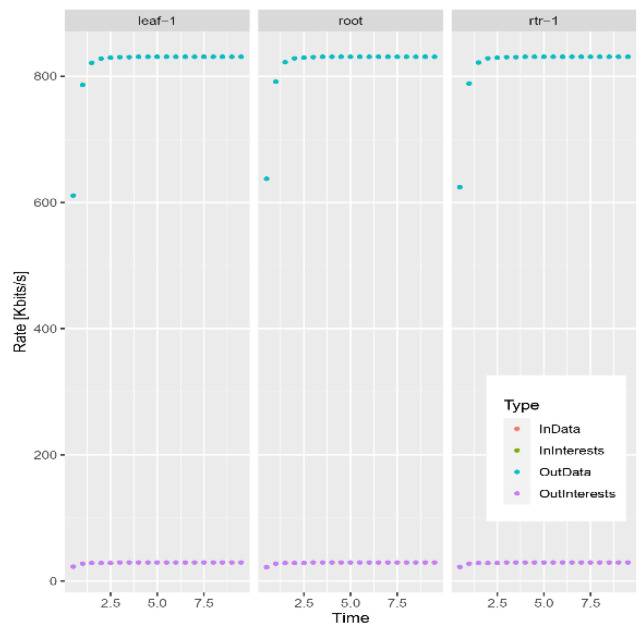
NDN network throughput.

**Figure 10:**
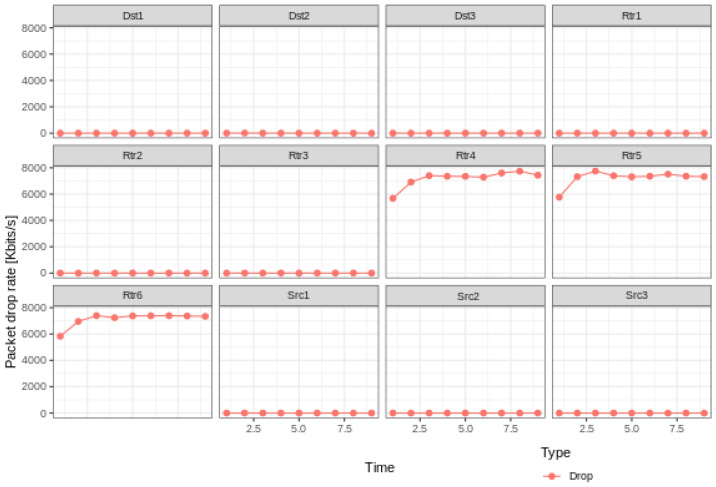
Packet loss rate of NDN network.

Because of the problems existing in the traditional blockchain network, such as mobility limitation, repeated confirmation transmission of data packets and security, the model replaces the underlying network of blockchain technology with an NDN and integrates the NDN technology with the blockchain to make up for the defects of traditional network and optimize network performance. To evaluate the model, this paper uses a convincing simulation platform in the industry to simulate the performance of traditional and NDN networks. In the experiment, the VIRTUAL machine Ubantu18.04 was used, and two simulation environments NS3 [11] and NDNSIM [12] were installed, respectively. In the simulation environment, the same two-node peer-to-peer network topology was constructed, respectively, and the data rate was set at 100 Mbps, as well as the same transmission delay of 10 ms. To be closer to the actual situation in the TCP communication experiment, a random error rate of 0.00001 was added to simulate the retransmission mechanism. In the experiment, all aspects of the performance of the two networks were compared. Figure 6, Figure 7 and Figure 8 show the operational data results of throughput, packet loss rate, and latency in 10 s, respectively, for the traditional network in the NS3 environment. Figure 9 and Figure 10 are the corresponding experimental results of the NDN network in the NDNSIM environment. The NDN network comparing traditional P2P networks and throughput experiments show that the forward load was more balanced among the nodes in the NDN network. The loss package rate experiment can be seen to be more stable to request nodes and release nodes in the NDN network, the situation being that the loss package is basically absent. The time delay experiment shows, in the NDN network, due to the advantages, such as reverse path sharing, high reuse rate of file routing sharing and named forward, the time delay will stabilize at a lower place. In the process of file sharing, path selection, low packet loss and low delay are important indicators to improve the reliability of file sharing. In summary, the NDN network has more optimal network performance and sharing reliability than the traditional blockchain underlying network, which also reflects the dominant performance of the model constructed in this paper that integrates the NDN network and blockchain technology.

### 4.2. Storage Efficiency

**Figure 11:**
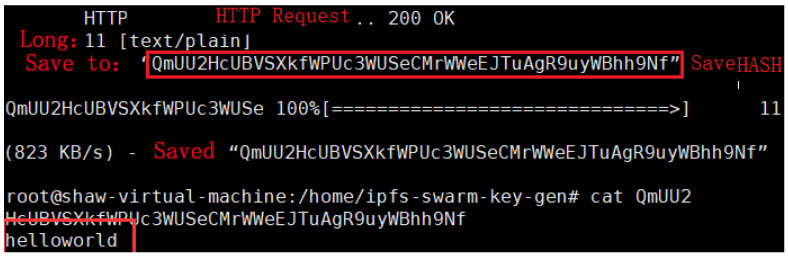
File download on the master node.

To evaluate the storage efficiency involved in the model, three Ubantu18.04 virtual machines in which the relevant test environments (such as Node.js, IPFS) were configured, respectively, were used in this paper. Finally, a local IPFS node was generated on all VMS, and the nodes were connected successfully to form a three-node private IPFS network. The private network included a master node system and two secondary node systems. The master node was responsible for adding files, and the secondary node could quickly download and view files. The experimental results are shown in Figure 11. It can be seen from the figure that a local node will obtain the corresponding hash value after the IPFS stores the file, and the corresponding file content can be seen only after another node requests the same hash value. The NDN needs to decrypt the file content again after obtaining the hash value.

### 4.3. Comprehensive Analysis

The existing file storage-sharing models can be divided into two categories. One is the traditional way, and some relevant models are quoted as follows: In [13], the authors proposed a solution to the degradation of file storage performance by minimizing the total amount of data stored in the network to meet the quality of service requirements of the solution. In [14], the authors proposed a point-to-point storage model considering user equity in which participating nodes shift their power of storage space upward and allocate storage space by judging contributions according to certain rules. In [15], the authors proposed a sharing model of distributed storage for large files, which provides the same access bandwidth regardless of the distance of the server. In [16], the authors proposed a peer-to-peer network file-sharing model to solve the problem of file loss caused by the process of joining or deleting nodes in peer-to-peer networks. The other is the blockchain solution, and part of the model is introduced as follows: [17] proposes a blockchain-based network resource sharing scheme to solve the reliable availability of electronic resource-sharing platforms. Table 2 compares the proposed model with the above model from the following dimensions: in terms of security, our model can meet the requirements without introducing an additional security mechanism or system level security protection. Under the action of the blockchain and the IPFS, it realizes the safe and efficient storage of files. In addition, it further protects the security of file storage procedures by combining file naming, signature and encryption with the NDN. Finally, through experimental proof and theoretical analysis, in view of its flexible routing strategy, reverse path forwarding and routing cache, the NDN network also improves the overall performance of the network and enhances the reliability in the process of file sharing and shows that the proposed model has more advantages in overall network performance, access control and storage.

### 4.4. Limitation

At present, the results of the study remain in the preliminary stage for normal size files. Aiming at the limitation of not being able to share in the real environment at present, a relatively mature and representative platform was selected in the research, and a simulation environment was successfully constructed. Experiments of file storage sharing of various sizes were tried to verify the reliability of the method model. In addition, there is a lack of existing studies on the integration of blockchain technology and other technologies in this field, and sufficient comparative consideration cannot be made. Therefore, this paper also tries to analyze the macro-performance.

## 5. Conclusions

In the process of knowledge creation, the massive growth of knowledge files leads to the problem of file storage and sharing. This model designs an NDN-based blockchain knowledge file storage and sharing model. Based on the private blockchain structure, the model utilizes the tamper-proof and traceability characteristics of blockchain-distributed storage and privately distributed storage IPFS to realize the secure and efficient storage of file contents. In addition, drawbacks, such as mobility problems of shared files in traditional blockchain systems, double confirmation of transmission and convergence with blockchain technology, were exploited to improve the overall network performance of the model and make the file-sharing process more efficient. This model effectively solves the problem of secure and efficient storage of knowledge files and improves the reliability of the file sharing process through the NDN network, but there are still some problems such as the foundation of trusted nodes of the private chain. Therefore, one direction in the future is to examine how to further ensure node reliability and file security when any user can log into the blockchain environment through registration. Finally, NDN technology is not mature enough, component development is not complete and application implementation is not complete. Moreover, there is still an unstable and imperfect situation. The optimization and guarantee of NDN and blockchain technology junction will also become the next research difficulty.

## 6. Patents

We have applied for two invention patents, including “data synchronization method, device and storage medium of blockchain node”, No.: 202110604013 X; “Optimization method, device and related equipment of dynamic routing and forwarding strategy”, No.: 202110601048.8.

## Figures and Tables

**Table 1:** Blockchain system architecture.

Layer	Conclusion
Data layer	The transaction data and code received by the node are packaged into time-stamped blocks of data linked to the current longest blockchain
Network layer	Including the blockchain system networking mode, message transmission protocol, data verification mechanism, etc.
Consensus layer	Solve the problem of how to effectively reach consensus in distributed systems

**Table 2:** Model performance comparison.

Model Contrast	Secure Storage	Smart Contract	IPFS	Forwarding Efficiency	Network Performance
S**SS**	Yes	No	No	No	No
Content Espresso	No	No	No	Yes	No
DSF	Yes	No	No	Yes	No
E-resource	Yes	Yes	Yes	Yes	No
Our Model	Yes	Yes	Yes	Yes	Yes

## Data Availability

Not applicable.
